# Prospective study to assess the survival outcomes of planned irradiation of ipsilateral subventricular and periventricular zones in glioblastoma

**DOI:** 10.3332/ecancer.2020.1021

**Published:** 2020-03-26

**Authors:** Deepthi Valiyaveettil, Monica Malik, Kothwal Syed Akram, Syed Fayaz Ahmed, Deepa M Joseph

**Affiliations:** 1 Department of Radiation Oncology, Nizam’s Institute of Medical Sciences, Punjagutta, Hyderabad 500082, India; 2 Department of Radiation Oncology, Yashoda Superspeciality Hospital, Malakpet, Hyderabad 500036, India; 3 Department of Radiation Oncology, All India Institute of Medical Sciences, Rishikesh, India

**Keywords:** glioblastoma, subventricular zone, periventricular zone, radiation

## Abstract

**Purpose/objective(s):**

Retrospective evidence suggests that the irradiation of stem cells in the periventricular zone (PVZ), specifically the subventricular zone (SVZ), to higher doses may be associated with improved outcomes.

**Materials/methods:**

This was a prospective study, done from 2012 to 2017 in glioblastoma patients to assess the efficacy of planned irradiation of ipsilateral PVZ and SVZ on survival outcomes. The clinical target volume included the tumour bed with a 1.5–2 cm margin, perilesional oedema and was expanded to encompass the ipsilateral PVZ (5 mm lateral expansion adjacent to the ventricles, including the SVZ, which was a 5 mm expansion lateral to lateral ventricle). The ipsilateral PVZ was planned to receive a dose of ≥50 Gy.

**Results:**

89 patients were recruited of which 74 patients were available for the analysis. Median age was 48 years. Mean doses to ipsilateral PVZ and SVZ were 56.2 and 55.1Gy, respectively. Median overall survival in the entire group was 13 months. There was no significant correlation between survival and doses to ipsilateral, contralateral, or bilateral PVZ and SVZ. Median survival was 16, 12 and 6 months for Eastern Cooperative ­Oncology Group (ECOG) PS 1, 2 and 3, respectively (*p* = 0.05).

**Conclusion:**

Planned irradiation of potential stem cell niches in the ipsilateral cerebral hemisphere did not result in improved survival as suggested by retrospective studies. Doses to contralateral or bilateral PVZ or SVZ also did not influence survival.

## Introduction

Glioblastoma is the most common malignant brain tumour [1]. Despite deeper insights into molecular biology and advances in therapeutics, the outcome remains poor for this aggressive tumour [2, 3]. The median overall survival (OS) is in the range of 14.6 to 21.1months [4, 5].

Recent research in glioblastoma demonstrated that the heterogeneity in survival outcomes and recurrence patterns may be related to neuronal stem cells (NSC) [6, 7]. NSCs reside in two areas in the adult mammalian brain: the subventricular zone (SVZ) and the subgranular zone (SGZ) [8] These have properties of self-renewal and multilineage potency along with tumour initiation and propagation [9, 10]. Hence, eliminating these cancer stem cells may prove useful for cure in gliomas [10].

Several retrospective studies have been done to assess the impact of incidental radiation dose to SVZ during the course of standard chemoradiation (CRT) on survival in high grade gliomas but with conflicting results [11–18]. The aim of our study was to prospectively analyse the effect of SVZ and periventricular zone (PVZ) irradiation on survival in glioblastoma.

## Materials and methods

Patients with histopathological diagnosis of primary glioblastoma, planned for adjuvant CRT at our institute from Jan 2012 to June 2017, were included in this prospective study. Following Institutional Review board approval patients of glioblastoma were prospectively recruited and informed consent was taken. All patients had undergone maximal safe resection. Patient demographics, clinical history and examination, radiological imaging and treatment details were documented. The details of extent of resection were collected from neurosurgeons’ treatment records and post-operative imaging.

### Radiotherapy planning

A thermoplastic immobilisation mask was done and CT simulation scans with 3 mm axial slices were acquired in treatment position. Pre and postoperative magnetic resonance imaging (MRI) images were co-registered with simulation scans. The gross tumour volume (GTV) was contoured as the contrast enhancing lesion on T1-weighted MRI or the post-operative cavity. The clinical target volume was a 1.5–2 cm margin around the GTV which was expanded to include the ipsilateral SVZ and PVZ, oedema and adjusted to anatomic barriers. The planning target volume was generated with a 5-mm isotropic margin expansion. The SVZ was contoured as a 5 mm lateral expansion of the lateral ventricles as per published guidelines [19]. The PVZ was contoured as 5 mm expansion all around the lateral ventricles. According to the spatial relationship with the tumour location SVZ and PVZ were classified as ipsilateral (i), contralateral (c) and bilateral (b). According to the spatial relationship with SVZ the tumours were classified as contacting SVZ and non-contacting SVZ tumours. Planned dose of adjuvant radiotherapy (RT) was 60 Gy in 30 fractions, 2 Gy per fraction, over 6 weeks with dynamic Multi-Leaf Collimator Intensity Modulated Radiotherapy (MLC IMRT) technique. A cone down boost volume was planned after 46 Gy if doses to critical structures exceeded tolerance levels. The ipsilateral SVZ and PVZ was planned to receive a mean dose of 50 Gy or more. All patients were planned for chemotherapy with TMZ, 75mg/m^2^ PO daily throughout the course of radiation followed by 150–200 mg/m^2^ PO for 5 days every 4 weeks for 6–12 cycles.

### Dosimetry

The plans were generated with Eclipse ^TM^ version 8.6 (Varian Medical Systems) treatment planning system. Dose-volume parameters for each SVZ and PVZ volume were extracted from the treatment plans. Dose volume histograms were generated and mean dose to the SVZ and PVZ structures were documented.

### Statistics

Data are presented as mean plus standard deviations (SD) and median for continuous variables. Categorical variables are presented as frequencies (%). The 25th, 50th and 75th percentiles were calculated for the dose to the iSVZ, cSVZ, bSVZ, iPVZ, cPVZ and bPVZ. Mean SVZ and PVZ doses were dichotomised at their median value at their median value into high dose and low dose arm and univariate analysis was done.

Survival times were calculated from the date of surgery and were estimated by the Kaplan–Meier methods with 95% confidence intervals (CI). Progression free survival (PFS) was defined as the time relapsed from date of surgery until the date of radiological or clinical evidence of progression or recurrence or patient death (if death occurred prior to radiological evidence of disease progression). Survival curves were compared using log rank test. Cox regression analysis was done for multivariate analysis.

Only variables with significance at or below the *p* = 0.05 level were considered as significant. All statistical analysis was done using SPSS v20 software (IBM, Chicago, IL, USA).

## Results

89 patients with primary glioblastoma were recruited for the study. Patients who did not complete the prescribed treatment and those who were lost to follow up (*n* = 15) were excluded from the analysis. Thus, a total of 74 patients were included in the final analysis. Patient characteristics are tabulated in Table 1.

The most common presenting complaint was headache (62%) followed by hemiparesis (24%). Other presentation symptoms were seizures, vomiting, fall, blurring vision, altered behaviour and memory disturbance. Mean duration of symptoms before surgery was 3.2 months. Tumours were grouped based on their relationship with SVZ as suggested by Jafri *et al* [6], and 62% of the cases were categorised as Group I. 70% of the tumours were contacting the SVZ. Other patient and treatment related characteristics are documented in Tables 1 and 2, respectively. The mean and median doses to iSVZ were 56.2 and 58 Gy, respectively. Dosimetric data are tabulated in Table 3.

### Survival

**Figure 1.**
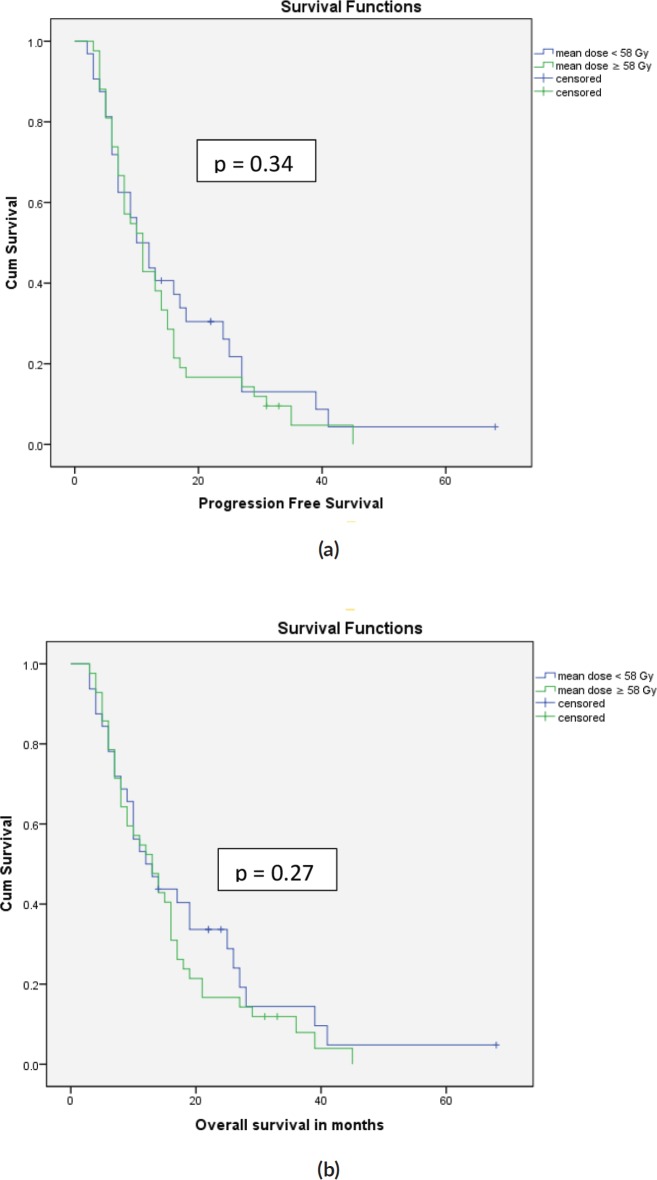
(a): Progression free survival curves for iSVZ mean doses <58 Gy versus ≥58 Gy. (b): Overall survival curves for iSVZ mean doses <58 Gy versus ≥58 Gy.

Median OS was 13 months and median PFS was 11 months (Figure 1a and b). Univariate analysis of prognostic factors showed significantly higher in survival in patients with better performance status (*p* = 0.05) and patients who received adjuvant chemotherapy (*p* = 0.00). Patients were divided into subgroups based on whether they received lower or higher than the median dose to ipsilateral subventricular zone (iSVZ) and iPVZ. Univariate analysis of prognostic factors in the higher and lower dose groups are given in Table 4. On comparing the lower and higher dose groups based on the median dose received by the ipsilateral, contralateral and bilateral SVZ and PVZ dose there was no significant difference in survival outcomes (Table 5).

On multivariate analysis, Eastern Cooperative Oncology Group (ECOG) performance status and concurrent and adjuvant temozolomide showed significant survival benefit (Table 6).

## Discussion

Glioblastoma is an aggressive and highly invasive primary CNS malignancy. Recent studies demonstrated that Glioblastomas contacting the SVZ are associated with earlier recurrence and poor survival [6, 20, 21].

Given the potential clinical significance of the stem cell niches in the SVZ, the concept of irradiation of SVZ seems logical to eliminate the CSCs. Many studies attempted to correlate incidental dose delivered to the SVZ with survival outcomes. These studies divided the patient population into a low SVZ dose and a high SVZ dose group and compared survival [22].

### Evidence supporting improved survival with higher SVZ and PVZ doses

The first study testing this hypothesis [11] was a retrospective study of 55 patients with high-grade glial tumours treated uniformly with surgery, conformal radiotherapy and chemotherapy. Patients were divided into groups based on the median bilateral periventricular dose of 43 Gy into a high dose (*n* = 28) and a low-dose group (*n* = 27). Despite containing a higher proportion of biopsy-only and subtotal resection (STR) patients, the high dose group (≥ 43 Gy) demonstrated significant improvement in PFS (median PFS 15 months versus 7.2 months; *p* = 0.03). Higher radiation dose to the PVZ was associated with decreased risk of progression for patients (RR: 0.74; *p* = 0.019, 95% CI: 0.567–0.951). The author summarised that a high dose of radiation of the PVZ is an independent predictor of PFS in glioblastoma.

Gupta *et al* [14] reported on a retrospective study of 40 patients with glioblastoma treated to 60 Gy in 30 fractions. On Cox regression analysis, increasing mean dose to the iSVZ was associated with significantly improved OS [HR = 0.87, 95%CI = (0.77; 0.98), *p* = 0.02]. This study results showed that iSVZ dose was an independent predictor of improved OS.

Lee *et al* [13] reported on a pooled analysis of 173 patients treated to 60 Gy in 30 fractions. They used 59.4 Gy as cut-off as they reasoned that a high dose would be required to kill radioresistant cancer stem cells. An iSVZ dose above 59.4 Gy was associated with statistically significant improvement in PFS on both univariate (HR 0.56; 95 % CI 0.32–0.98; *p* = 0.042) and multivariate analysis (HR 0.45; 95 % CI 0.25–0.82; *p* = 0.009) but no benefit in OS. When we used the same cut off in our study, it did not show significant results (PFS 10 months versus 11 months, *p* = 0.92).

Iuchi *et al* [23] reported on a prospective phase II trial of 46 patients with Glioblastoma treated with hypofractionated radiotherapy and concomitant chemotherapy with temozolomide. Median OS was 20.0 months and the results showed that radiation necrosis in the SVZ strongly correlated with improved survival but caused deterioration in the performance status of long-term survivors. Median OS was 36.2 months in patients with SVZ necrosis versus 13.3 months without necrosis (HR 4.08; 95 % CI 1.97–9.10, *p* = 0.0001). In multivariate analysis only SVZ necrosis was significantly associated with prolonged survival.

Another retrospective study of 43 patients with glioblastoma [24] was conducted to identify SVZ related prognostic factors for survival and recurrence patterns. Results on multivariate analysis showed contact to SVZ, as well as reduced bSVZ radiation dose coverage (V20Gy <84%), might be independent poor prognostic factors for time to progression (7 months versus 5.2 months, *p* = 0.017).

Interim analysis of our study data (54 patients) investigating planned neural stem cell niche irradiation in glioblastoma [25] showed mean dose of 58 Gy or greater to the iSVZ correlated positively with improved OS (16 months versus 14 months, *p* = 0.03).

A recent retrospective study [26], including 47 patients with glioblastoma, showed a non-significant trend towards improved survival at a median follow up of 19 months with mean dose higher than 56 Gy to the ipsilateral SVZ.

### Evidence showing no correlation of survival with SVZ or PVZ doses

Researchers from the Netherlands [12], however, could not replicate these results. They analysed 87 patients with newly diagnosed glioblastoma treated with standard trimodality therapy. Using a cutoff of 43 Gy (threshold from Evers *et al*), there was no correlation between ipsilateral, contralateral or bilateral SVZ dose with PFS or OS. Threshold values of 30, 40 or 50 Gy also could not demonstrate any correlation between SVZ dose and survival. However, in the subgroup with complete resection (*n* = 32), using a cutoff 30 Gy mean dose for bSVZ, there was a statistically significant correlation between dose and OS (*p* = 0.015). In our study, we did not find any significant PFS benefit with dose higher than 43 Gy or 30 Gy to bSVZ.

A larger study at Johns Hopkins University, by Chen *et al*, [15] reported on a retrospective series of 116 patients with Glioblastoma treated to 60 Gy in 30 fractions with concurrent chemotherapy. In this study, patients receiving a high dose (>40 Gy) to the iSVZ did not have significantly different survival outcomes to the low dose group. In 41 (35 %) of patients undergoing a gross total resection (GTR), the authors reported improved PFS (15.1 versus 10.3 months, *p* = 0.028, HR 0.385, 95 % CI0.165–0.901) and OS (17.5 versus 15.6 months, *p* = 0.027, HR 0.385, 95 % CI 0.165–0.895) for patients who underwent GTR and received a higher dose (>40 Gy) to the iSVZ. Recent re-analysis of 102 patients [27] from this cohort did not find any significant correlation between SVZ radiation dose and distant recurrence, although this analysis included even the subtotal and biopsy only patients along with GTR patients. In our study 21 patients (28%) underwent gross total resection and we found no correlation with PFS or OS in this group.

A study by Chua *et al* [28] evaluated if dose escalated hypofractionated RT (IMRT with SIB, 70 Gy in 30 fractions) improves tumour control and survival outcomes in patients with Glioblastoma. Mean doses (Gy) were 60.6 (range 33.4–69.8), 39.5 (19.4–61.2), 49.1 (28.3–64.3) to the ipsilateral, contralateral, and composite SVZ, respectively. There was no positive association between SVZ doses and PFS (HR = 1.05, ipsilateral, 95% CI = 0.88–1.24; 1.143, contralateral, 0.94–1.39; 0.80, composite, 0.57–1.12).

A study done by Murchison *et al* [29] included a large cohort of 370 glioblastoma patients. This study had a median follow up of 16.4 months. Poor KPS, biopsy only, multifocality and no adjuvant chemotherapy was observed as poor prognostic factors for survival. It showed no improvement in survival with increasing dose to SVZ.

A recent systematic review and meta-analysis [30] was published which compared high versus low doses. They observed that higher dose of irradiation to the ipsilateral SVZ showed statistically significant improvement in PFS compared to lower doses. These results, however, did not translate in OS.

### Evidence suggesting detrimental effect on survival with higher doses to SVZ and PVZ

Elicin *et al* [16] reported contradictory results showing high iSVZ dose of >62.25 Gy did not improve PFS in even in good performance status [HR: 2.58 (95% CI 1.03–6.05), *p* = 0.044] and tumours not contacting SVZ [HR: 10.57 (95% CI 2.04–49), *p* = 0.008] groups.

A recent study [31] conducted at Tata Memorial Hospital, India, included 80 patients with glioblastoma and showed that patients who received <59 Gy to the iSVZ had significantly better outcomes than patients who received greater than 59 Gy to the iSVZ both in terms of median PFS (20.5 versus 9.7 months, *p* = 0.016) and OS (20.6 vesrus 13.3 months, *p* = 0.026).

### SVZ and PVZ doses and neurocognitive effects

In the scenario of longer survival neurocognitive toxicity of radiation is one of the important concerns which can result due to damage to neural progenitor cells (NPCs). Redmond *et al* [32] in their study in mice and human models demonstrated that NPC sparing clinical plans may reduce neurocognitive effects. A prospective study in 30 patients showed inverse relationship between RT dose to the NPC niches and neurocognitive effects in patients with no effects on recurrence or survival [33]. A recent retrospective analysis comparing the impact of sparing stem cell with IMRT and non IMRT plans showed no significant difference in the OS, concluding that there was no impact on sparing stem cells on survival.

Many of the studies which demonstrated a positive correlation of doses to the SVZ and PVZ with survival were retrospective and analysed the incidental coverage of SVZ in treatment fields. Heterogeneity in the patient characteristics and dose cut offs at which statistically significant relationships are observed limit the validity of the conclusions drawn. There is conflicting data regarding the benefit of higher dose to SVZ on survival. Prospective data including ours failed to demonstrate any improvement in survival outcomes. Recent data has shown worse outcomes with higher SVZ doses. The detrimental effect on neuro-cognition with higher doses to NSCs is also of concern. Thus, there is no strong evidence to suggest higher dose to SVZ or PVZ improves survival it will be prudent to spare these areas and reduce neurocognitive decline.

### Limitations

he study did not evaluate or stratify for other established prognostic factors in Glioblastoma, including MGMT methylation, IDH1 mutation, etc. Neurocognitive function was not assessed in this study.

## Conclusion

Increasing dose to the iSVZ and PVZ did not translate into improved survival outcomes in patients of Glioblastoma. Future studies should focus on sparing of these areas to preserve neurocognitive function*.*

## Funding declaration

No funding was received for the study.

## Conflict of interest statement

All authors declare no conflict of interest.

## Figures and Tables

**Table 1.** Patient characteristics

Patient variable (*N* = 74)	Number of patients (%)
Age in years	Range—19–79 Median—48 Mean—47.2 (SD ± 13.5)
Sex Male Female	57 (77%) 17 (23%)
Common clinical presentation Headache Unilateral weakness Seizures Vomiting Memory disturbance Altered Behaviour Visual disturbances	43 (58%) 16 (21%) 15 (20%) 11 (15%) 7 (9%) 6 (8%) 5 (7%)
Mean duration of symptoms	3.2 months(0.1 – 12 months)
Performance status ECOG PS 1 ECOG PS 2 ECOG PS 3	27 (36%) 31 (42%) 16 (22%)
Grouping according to relationship to SVZ [6] I II III IV	46 (62%) 6 (8%) 17 (23%) 5 (7%)
Tumour contacting SVZ Yes No	52 (70%) 22 (30%)

ECOG PS, Eastern Cooperative Oncology Group Performance Status; SVZ, Sub ventricular zone

**Table 2.** Treatment details.

Patient variable (*N*= 74)	Number of patients (%)
Extent of Surgery Gross total excision Subtotal excision Biopsy	21 (28%) 49 (66%) 4 (6%)
RT technique 3D conformal technique Intensity modulated RT	41 (56%) 33 (44%)
Concurrent temozolomide Yes No	66 (89%) 8 (11%)
Adjuvant temozolomide Yes No	47 (63%) 27 (37%)

3D, three dimensional; RT, radiotherapy

**Table 3.** Doses received by the SVZ and PVZ.

Zone	Mean dose in Gray± SD	Median dose in Gray
iSVZ	56.2± 7.3	58.0
cSVZ	44.5± 9.2	46.3
bSVZ	49.6± 7.4	51.4
iPVZ	55.1± 7.7	57.5
cPVZ	45.1± 9.0	46.0
bPVZ	49.5± 7.5	51.0

SVZ, subventricular zone; PVZ, periventricular zone; I, ipsilateral; C, contralateral; B, bilateral

**Table 4:** Prognostic factors

Factor	PFS in months	*p*-value	OS in months	*p*-value
Age <50 years versus ≥50 years	13 versus 8	0.13	14 versus 10	0.17
ECOG PS 1 versus 2 versus 3	16 versus 10 versus 6	**0.009**	16 versus 12 versus 6	**0.05**
Extent of resection GTR versus STR versus Biopsy	12 versus 11 versus 6	0.97	13 versus 13 versus 6	0.93
RPA score I II versus IV versus V/VI	16 versus 9 versus 7	0.09	17 versus 10 versus 12	0.09
Group I versus II versus III versus IV	11 versus 6 versus 11 versus 10	0.60	13 versus 11 versus 11 versus 21	0.73
Tumours Contacting SVZ Yes versus No	10 versus 11	0.66	11 versus 13	0.67
Temozolomide Adjuvant Yes versus No	12 versus 8	**0.003**	15 versus 8	**0.00**

ECOG PS, Eastern Cooperative Oncology Group Performance Status; SVZ, Sub ventricular zone; GTR, Gross total resection; STR, Sub total resection; RPA, Recursive partitioning analysis

**Table 5.** PVZ and SVZ dose correlation with survival.

Variable	Dose (in Gray) groups	PFS in months	*p* value (log rank)	OS in months	*p* value (log rank)
iSVZ	<58.0 versus ≥58.0	12 versus 11	0.34	13 versus 13	0.27
cSVZ	<46.3 versus ≥46.3	10 versus 11	0.31	13 versus 12	0.15
bSVZ	<51.4 versus ≥51.4	10 versus 11	0.56	13 versus 12	0.33
iPVZ	<57.5 versus ≥57.5	12 versus 10	0.43	15 versus 11	0.22
cPVZ	<46.0 versus ≥46.0	9 versus 11	0.78	13 versus 12	0.83
bPVZ	<51.0 versus ≥51.0	10 versus 11	0.98	13 versus 12	0.38

SVZ, subventricular zone; PVZ, periventricular zone; i, ipsilateral; c, contralateral; b, bilateral

**Table 6.** Multivariate Cox Regression analysis of prognostic factors and survival.

Variable	Progression Free Survival			OS		
	HR	95% CI	p value	HR	95% CI	p value
Age (<50 versus ≥50 years)	1.00	0.98–1.03	0.50	1.01	0.98–1.03	0.37
ECOG Performance status (1 versus >1)	1.84	1.12–3.01	**0.01**	1.61	0.99–2.63	**0.05**
Tumors contacting SVZ (yes vs no)	0.93	0.53–1.63	0.80	0.82	0.47–1.42	0.48
Surgery (GTR versus < GTR)	1.45	0.59–3.57	0.40	0.22	0.71–4.15	0.22
Concurrent TMZ (yes versus no)	0.34	0.12–0.92	**0.03**	0.33	0.12–0.91	**0.03**
Adjuvant TMZ (yes versus no)	3.36	1.68–6.73	**0.00**	4.15	2.04–8.41	**0.00**
iSVZ (<58.0 Gy versus ≥58.0 Gy)	0.85	0.40–1.79	0.67	0.80	0.38–1.70	0.57

ECOG PS: Eastern Cooperative Oncology Group Performance Status; SVZ: Sub ventricular zone; i- ipsilateral, TMZ – Temozolomide; GTE – Gross total rescection
